# Predictive study of pharmacological reversal for residual neuromuscular blockade and postoperative pulmonary complications: a prospective, observational, cohort study

**DOI:** 10.1038/s41598-022-18917-y

**Published:** 2022-09-02

**Authors:** Cristian Aragón-Benedí, Ana Pascual-Bellosta, Sonia Ortega-Lucea, Sara Visiedo-Sánchez, Javier Martínez-Ubieto, Research Group in Anaesthesia, Resuscitation, and Perioperative Medicine of Institute for Health Research Aragón (ISS Aragón), Cristian Aragón-Benedí, Ana Pascual-Bellosta, Sonia Ortega-Lucea, Javier Martínez-Ubieto, Luis Alfonso Muñoz-Rodríguez, Guillermo Pérez-Navarro, Natividad Quesada-Gimeno, Lucía Tardós-Ascaso, Sara Visiedo-Sánchez, Teresa Jiménez-Bernadó, Berta Pérez-Otal, Francisco Romero-Caro

**Affiliations:** 1 Department of Anesthesia, Resuscitation and Pain Therapy, Miguel Servet University Hospital, 50009 Zaragoza, Spain; 2 Department of Anaesthesia, Resuscitation and Pain Therapy, University Clinical Hospital Lozano Blesa, Zaragoza, Spain; 3 Institute for Health Research Aragón (IIS Aragón), Zaragoza, Spain

**Keywords:** Risk factors, Respiratory tract diseases, Comorbidities, Respiratory signs and symptoms, Medical research

## Abstract

In recent years, some studies have generated controversy since they conclude that intraoperatively pharmacological reversal of neuromuscular blockade does not contribute to the reduction of postoperative residual neuromuscular blockade or pulmonary complications. Therefore, the main objective of this study was to assess the incidence of residual neuromuscular blockade and postoperative pulmonary complications according to spontaneous or pharmacological neuromuscular reversal. The secondary aim was to present a prognostic model to predict the probability of having postoperative residual neuromuscular blockade depending on a patient's comorbidities and intraoperative neuromuscular blocking agents management. A single-center, prospective, observational cohort study including patients undergoing surgical procedures with general anesthesia was designed. A total of 714 patients were analyzed. Patients were divided into four groups: cisatracurium with spontaneous reversal, cisatracurium with neostigmine antagonism, rocuronium with spontaneous reversal, and rocuronium with sugammadex antagonism. According to our binomial generalized linear model, none of the studied comorbidities was a predisposing factor for an increase in the residual neuromuscular blockade. However, in our study, pharmacological reversal of rocuronium with sugammadex and, particularly, neuromuscular monitoring during surgery were the factors that most effectively reduced the risk of residual neuromuscular blockade as well as early and late postoperative pulmonary complications.

## Introduction

More than 400 million people receive neuromuscular blocking agents annually to paralyze skeletal muscle groups, facilitate tracheal intubation, allow for controlled mechanical ventilation and achieve optimum relaxation conditions for surgery^1,2^.

The possibility of residual neuromuscular blockade (RNMB) after using neuromuscular blocking agents has been known for some time. However, in recent years, there has been an increase in the number of publications showing its high incidence, its relationship to postoperative pulmonary complications (POPC), and increased potential healthcare costs^3–6^. POPC include upper airway obstruction, oxygen desaturation, bronchoaspiration, pneumonia, atelectasis, and reintubation for severe respiratory failure requiring an unplanned admission to an intensive care unit (ICU)^2,3,5–7^.

Numerous studies and multiple international organizations have suggested that every patient receiving non-depolarising neuromuscular blocking drugs should have at least qualitative, and preferably quantitative intraoperative monitoring of the neuromuscular blockade (NMB) and assessment of the pharmacologic antagonism of NMB^8–11^. Nevertheless, anesthesia professionals have not widely utilized quantitative measurements of drug-induced NMB and the adequacy of pharmacologic reversal^3–5^. Furthermore, intraoperative neuromuscular monitoring (NMM) rates vary according to each center and do not cover the entire surgical patients with general anaesthesia and neuromuscular blocking agents^11–13^.

In addition, in recent years, some studies have generated controversy since they contradict most previous studies, concluding that intraoperative monitoring of the NMB and pharmacological reversal do not contribute in any way to the reduction of postoperative RNMB or pulmonary complications^2,14^.

In this regard, the primary objective of this study was to assess the incidence of RNMB and POPC according to spontaneous or pharmacological neuromuscular reversal, based on current clinical practice. The secondary objective was to present a prognostic model to predict the probability of having RNMB depending on the patient's comorbidities and the intraoperative management of the neuromuscular blocking agents.

## Methods

### Study design and setting

A single-center, prospective, observational cohort study was designed that included patients undergoing elective or emergency surgery at Miguel Servet University Hospital in Zaragoza from January 2016 to December 2019. The reporting of this study conforms to the STROBE statement.

### Ethics

The study was first approved by the Ethical and Research Committee of Miguel Servet University Hospital, Zaragoza, Spain, with registration code 06/2014 (Chairperson J.M. Larrosa Poves). Subsequently, it was reauthorized by the Regional Ethics Committee of Aragón (CEICA), with the number CAB-SUG-2019-01 (Chairperson M. Gonzalez Hinjos) as requested by regional guidelines. This study was performed in line with the principles of the Declaration of Helsinki and written informed consent was obtained from all subjects.

### Inclusion/exclusion criteria

The inclusion criteria were: patients with ASA physical status I to III, aged over 18 years, who were to undergo general anaesthesia with neuromuscular blocking agents and signed informed consent. Exclusion criteria included patients with ASA physical status IV to V, known neuromuscular disease, diabetes mellitus with diagnosed neuropathy, pregnancy or lactation, known allergy to neuromuscular blocking agents, cardiac surgery, or planned admission to surgical ICU with mechanical ventilation. The patients were selected before the surgery**,** having signed the consent form for inclusion in the study.

### Primary and secondary outcomes

The primary outcome was the presence of postoperative RNMB, defined as a TOF ratio < 0.9 at admission to the PACU.

The secondary outcomes were the POPC, as defined in other studies like ARISCAT^15^ or PERISCOPE^16^. Early POPC were considered as at least one of the following respiratory events in the PACU: upper airway obstruction, desaturation below 92%, bronchoaspiration, or need for reintubation for the severe respiratory failure of the patient. Late POPC were defined as at least one event of pneumonia or atelectasis in the 30 days following surgery.

### Patient population and anaesthesia

The recruited patients were those who were to receive neuromuscular blocking agents under balanced general anaesthesia. Neuromuscular blockade was performed according to routine clinical practice and usual department protocol with cisatracurium (0.1–0.2 mg/kg) or rocuronium (0.6–1.2 mg/kg) for anaesthetic induction at the choice of the anaesthesiologist in charge of the patient who was blinded to the patient’s inclusion in the study. Similarly, anaesthetic maintenance, intraoperative quantitative NMM, repeated doses of the neuromuscular blocking agent, or pharmacological reversal at the end of surgery depended on the clinical criteria of the same anaesthesiologist. If pharmacological antagonism was performed, patients with rocuronium received sugammadex (2–4 mg/kg), and those with cisatracurium were administered neostigmine (0.03–0.05 mg/kg) and atropine (0.02 mg/kg) according to routine clinical practice. In our department protocol, we did not assess the reversal of rocuronium with neostigmine, given the current evidence of increased postoperative complications with this combination^6,7^.

According to the neuromuscular blocking agents and spontaneous or pharmacological reversal, the patients were then categorized into four groups: group 1 cisatracurium without pharmacological reversal, group 2 cisatracurium with neostigmine antagonism, group 3 rocuronium without pharmacological reversal, and group 4 rocuronium with sugammadex antagonism.

### Measurements and data handling

Patient demographic data included age, weight, gender, ASA physical status, and comorbidities (chronic obstructive pulmonary disease, obstructive sleep apnea syndrome, restrictive lung disease, asthma, acute myocardial infarction, heart failure, high blood pressure, anaemia, chronic renal failure, diabetes mellitus, dyslipidemia, hyperthyroidism, hypothyroidism, chronic liver disease, dementia, and fragility).

The type of surgery (general surgery, maxillofacial surgery, otolaryngology, urology, vascular surgery, and others), emergency or elective procedure, intraoperative quantitative NMM during surgery, and repeated doses of the neuromuscular blocking agent were recorded as intraoperative data.

To measure the postoperative RNMB, we used, in 100% of the patients at admission to the PACU, a single TOF measurement (four stimuli of 0.2 ms in duration at a frequency of 2 Hz) with an intensity of 40 mA using a TOF-Watch-SX^®^ acceleromyography device [Organon, Oss, The Netherlands] calibrated in the operating room before the first dose of NMB. It was performed by the research staff, who was blinded and was not involved in the intraoperative care of the patient.

To assess the POPC, we consulted the patient’s electronic clinical history, recording any clinical event in PACU or on the hospital ward, laboratory test, radiological study, and primary care or emergency room consultation reports during hospital admission or 30 days after surgery confirming the type of POPC.

### Sample size

Assuming an incidence of residual neuromuscular blockade with rocuronium and cisatracurium of 13%^17^ and 34.1%^7^, respectively, with a significant level of 5% and 95% of power, a sample size of 103 patients was calculated using the EPIDAT v. 4.1. software. To account for dropouts**,** we included at least 110 patients per group. Patient recruitment was performed through a sequential review of cases in a recruitment period from January 2016 to December 2019.

### Statistical analysis

A descriptive analysis was completed to perform data analysis using the mean, standard deviation, and quartiles to summarize quantitative data according to normal distribution. For qualitative data, frequency and percentages were used. A χ^2^ test and a Fisher’s test were used for qualitative variables, and when proportions were compared for different groups, a difference in proportions test was used. A Kruskal–Wallis test and an analysis of variance (ANOVA) test were used to study the relationship of a qualitative variable with a quantitative variable.

A Binomial Generalized Linear Model was performed to predict RNMB using the demographic data (age, weight, gender, ASA), the comorbidities (chronic obstructive pulmonary disease, obstructive sleep apnea syndrome, restrictive lung disease, asthma, acute myocardial infarction, heart failure, high blood pressure, anaemia, chronic renal failure, diabetes mellitus, dyslipidemia, hyperthyroidism, hypothyroidism, chronic liver disease, dementia, and fragility) and the variables of the neuromuscular blockade management (neuromuscular blocking agent, intraoperative NMM, pharmacological reversal) as previously detailed.

For this, the Likelihood-Ratio test was used to select the variables of the Binomial Generalized Linear Model with Logistic Regression (logit link) that were part of the final model. The modeling process was carried out in stages, eliminating the variables with a lower significance or equivalently with a higher p-value for the Likelihood-Ratio Test in each stage.

Differences for which the p-value was < 0.05 were considered significant. The analysis has been developed with R version 3.4.4 (R Foundation for Statistical Computing, Vienna, Austria). The statistical analysis and the data review were developed by Jorge Luis Ojeda Cabrera Ph.D. (Dept. of Statistical Methods of the University of Zaragoza).

## Results

**Figure 1:**
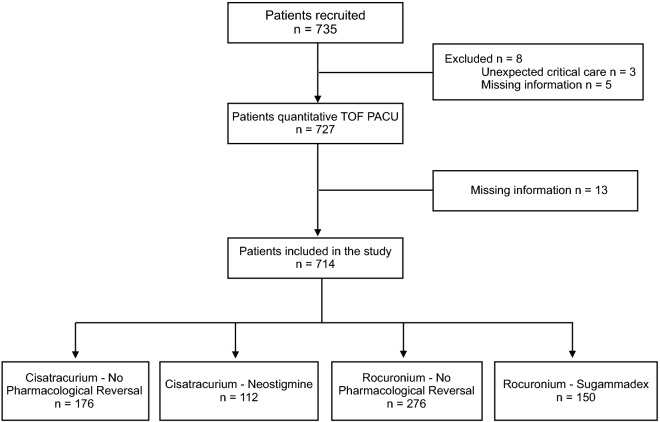
STROBE patient flow diagram. TOF, train of four; PACU, post-anaesthesia care unit.

During the study period, 735 patients were included, 21 of whom were excluded**,** as detailed in Fig. 1. STROBE patient flow diagram. Patients were divided into the four groups detailed in the methodology by type of neuromuscular blocking agent and spontaneous or pharmacological reversal (Fig. 1). The groups were homogeneous, and there were no differences between the groups in patient demographic data or comorbidities (Table 1).

**Table 1:** Homogeneity and comparison of demographic data and comorbidities between groups.

Quantitative variables (n)	Cisatracurium—no reversal group n = 176		Cisatracurium + neostigmine group n = 112		Rocuronium—no reversal group n = 276		Rocuronium + sugammadex group n = 150		P-value
	Mean	SD	Mean	SD	Mean	SD	Mean	SD	ANOVA
Age; years (714)	59.8	17.3	60.8	16.7	60.7	16.3	59.4	15.5	0.81
Weight; kg (714)	70.2	11.9	72.6	12.5	72.1	13.2	75.4	16.8	0.11
ASA Score (714)	2.10	0.69	2.16	0.71	2.11	0.67	2.14	0.66	0.89

Qualitative variables (n)	Percent % (n)	Percent % (n)	Percent % (n)	Percent % (n)	χ^2^ test				
Male (474)	23.2% (110)	16.2% (77)	40.1% (190)	20.4% (97)	0.49				
Female (240)	27.5% (66)	14.5% (35)	35.8% (86)	22.1% (53)					
COPD (85)	21.1% (18)	16.4% (14)	49.4% (42)	12.9% (11)	0.09				
OSAS (32)	40.6% (13)	3.10% (1)	34.3% (11)	21.8% (7)	0.07				
Restrictive lung disease (8)	12.5% (1)	25.0% (2)	25.0% (2)	37.5% (3)	0.49				
Asthma (14)	35.7% (5)	0.00% (0)	42.8% (6)	21.4% (3)	0.38				
AMI (67)	22.3% (15)	23.8% (16)	35.8% (24)	17.9% (12)	0.28				
Heart failure (20)	20.0% (4)	15.0% (3)	40.0% (8)	25.0% (5)	0.94				
High blood pressure (329)	26.4% (87)	17.6% (58)	37.9% (125)	17.9% (59)	0.16				
Anaemia (62)	30.6% (19)	17.7% (11)	35.4% (22)	16.1% (10)	0.54				
Chronic renal failure (40)	37.5% (15)	17.5% (7)	27.5% (11)	17.5% (7)	0.20				
DM (131)	23.6% (31)	21.3% (28)	36.6% (48)	18.3% (24)	0.25				
Dyslipidemia (165)	24.2% (40)	18.7% (31)	37.5% (62)	19.3% (62)	0.64				
Hyperthyroidism (4)	0.00% (0)	25.0% (1)	50.0% (2)	25.0% (1)	0.71				
Hypothyroidism (2)	27.2% (6)	13.6% (3)	45.4% (10)	13.6% (3)	0.80				
Chronic liver disease (20)	35.0% (7)	20.0% (4)	20.0% (4)	25.0% (5)	0.36				
Dementia (2)	0.00% (0)	0.00% (0)	0.00% (0)	100% (2)	0.05				
Fragility (114)	20.1% (23)	17.5% (20)	43.8% (50)	18.4% (21)	0.42				

Basic descriptives and tests for the demographic and comorbidity variables for each group. As can be seen, there was no significant relationship between the demographic and comorbidity variables and each group. Absolute (N) and relative (%) frequencies along with independence tests (χ^2^) for the qualitative variables, and mean and standard deviation (SD) along with comparing means tests (analysis of variance [ANOVA]) for the quantitative variables. *Significance defined as p-value < 0.05.

ASA, American Society of Anesthesiologists score; COPD, chronic obstructive pulmonary disease; OSAS, obstructive sleep apnea syndrome; AMI, acute myocardial infarction; DM, diabetes mellitus.

### Residual neuromuscular blockade between groups

We found that 28.3% (n = 202) of all patients had RNMB. According to the four groups, the incidence of RNMB was: group 1 cisatracurium without pharmacological reversal 33.52% (n = 59), group 2 cisatracurium with neostigmine antagonism 30.35% (n = 34), group 3 rocuronium without pharmacological reversal 35.87% (n = 99), and group 4 rocuronium with sugammadex antagonism 5.33% (n = 8), with p < 0.001, χ^2^ test (Table 2).

**Table 2:** Incidence of residual neuromuscular blockade, early and late postoperative pulmonary complications between groups.

Qualitative variables	Cisatracurium—no reversal group n = 176	Cisatracurium + neostigmine group n = 112	Rocuronium—no reversal group n = 276	Rocuronium + sugammadex group n = 150	P-value
	Percent % (n)	Percent % (n)	Percent % (n)	Percent % (n)	χ^2^ test
RNMB	33.5% (59)	30.3% (34)	35.8% (99)	5.33% (8)	< 0.001*
Early POPC	26.7% (47)	18.7% (21)	12.3% (34)	4.67% (7)	< 0.001*
Late POPC	7.39% (13)	8.93% (10)	9.78% (27)	2.67% (4)	0.038*

Absolute (N) and relative (%) frequencies for each group along with independence tests (χ^2^) *Significance defined as p-value < 0.05.

RNMB, residual neuromuscular blockade; POPC, postoperative pulmonary complications.

### Intraoperative neuromuscular monitoring and residual neuromuscular blockade

Intraoperative NMM was used in 30.3% (n = 216) of patients, with no statistically significant differences in the four groups (p = 0.98, χ^2^ test).

If we analyze the influence of intraoperative NMM and RNMB, patients not monitored intraoperatively had an incidence of RNMB of 35.7% (n = 178). However, when monitored, the incidence decreased to 10.2% (n = 22) with p < 0.001, χ^2^ test (Table 3).

**Table 3:** Incidence of residual neuromuscular blockade and postoperative pulmonary complications if exists both intraoperative neuromuscular monitoring and pharmacological reversal.

Qualitative variables	No intraoperative NMM	Intraoperative NMM	P-value	No pharmacological reversal	Pharmacological reversal	P-value
	Percent % (n)	Percent % (n)	χ^2^ test	Percent % (n)	Percent % (n)	χ^2^ test
RNMB	35.7% (178)	10.2% (22)	< 0.001*	35.0% (158)	16.0% (42)	< 0.001*
Early POPC	17.2% (86)	10.6% (23)	0.023*	17.9% (81)	10.7% (28)	0.009*
Hypoxaemia	12.2% (61)	7.87% (17)	0.084	12.8% (58)	7.63% (20)	0.031*
Airway obstruction	5.02% (25)	2.78% (6)	0.176	5.09% (23)	3.03% (8)	0.198
Late POPC	9.24% (46)	3.70% (8)	0.011*	8.85% (40)	5.34% (14)	0.087
Pneumonia	2.01% (10)	0.92% (2)	0.301	1.99% (9)	1.15% (3)	0.396
Atelectasis	7.63% (38)	3.70% (8)	0.049*	7.30% (33)	4.96% (13)	0.219

Absolute (N) and relative (%) frequencies for each group along with independence tests (χ^2^) *Significance defined as p-value < 0.05.

RNMB, residual neuromuscular blockade; POPC, postoperative pulmonary complications; NMM, neuromuscular monitoring.

### Postoperative pulmonary complications between groups

Concerning the respiratory events, a total of 15.27% (n = 109) of all patients had some early POPC in the PACU. Of the total patients, 10.92% (n = 78) presented oxygen desaturation and 4.34% (n = 31) presented upper airway obstruction. There were no cases of bronchoaspiration or reintubation for severe respiratory.

On the other hand, the incidence of late POPC at 30 days after surgery was 8.12% (n = 58): 6.44% (n = 46) had atelectasis and 1.68% (n = 12) had pneumonia (Tables 2 and 3).

### Predictive model for residual neuromuscular blockade

All those variables with a positive coefficient estimate contributed to increasing the incidence, while those with a negative coefficient decreased it (Table 4). None of the demographic data and comorbidities added to the model predisposed to having more RNMB. However, the type of neuromuscular blocking agent used, TOF monitoring during surgery, and pharmacological reversal did have a significant effect.

**Table 4:** Variables and coefficients of the generalized linear model with likelihood-ratio test to predict residual neuromuscular blockade.

	Estimate	Std. error	Z value	Pr ( >|z|)
(Intercept)	− 0.08	0.15	− 0.56	0.57
Rocuronium	− 0.44	0.17	− 2.50	0.010*
Intraoperative NMM	− 1.46	0.24	− 5.91	< 0.001*
Sugammadex	− 0.88	0.20	− 4.33	< 0.001*

Significant coefficients of the Generalized Linear Model along with the standard error (Std. Error), the corresponding Z value and p-values [Pr ( >|z|)]. The sign of the coefficients of each variables indicates the direction of the influence in the residual neuromuscular blockade. In our case, all the coefficients were negative, so they were factors that reduced the probability of the residual neuromuscular blockade; *Significance defined as p-value < 0.05.

NMM, neuromuscular monitoring.

Specifically, as can be seen from the following data (Table 4), the pharmacological combination of rocuronium (− 0.44, coefficient estimate) with sugammadex (− 0.88, coefficient estimate) and, particularly, intraoperative NMM (− 1.46, coefficient estimate) significantly reduced the incidence of RNMB.

When using rocuronium, avoidance of intraoperative NMM and neuromuscular blockers antagonism led to an incidence of RNMB of 41.27%; Conversely, the use of monitoring and pharmacological reversal decreased the probability to 2.17% (Table 5).

**Table 5:** Probability of residual neuromuscular blockade according to the neuromuscular blocking agent, neuromuscular monitoring and pharmacological reversal according to the generalized linear model.

NMB agent	Intraoperative NMM	Pharmacological reversal	Probability RNMB (%)
Cisatracurium	No	No	40.5
Rocuronium	No	No	41.2
Cisatracurium	Yes	No	14.2
Rocuronium	Yes	No	14.6
Cisatracurium	No	Neostigmine	39.5
Rocuronium	No	Sugammadex	8.33
Cisatracurium	Yes	Neostigmine	13.7
Rocuronium	Yes	Sugammadex	2.17

NMB, neuromuscular blockade; NMM, neuromuscular monitoring; RNMB, residual neuromuscular blockade.

## Discussion

This prospective, observational cohort study was intended to clarify certain questions arising in recent years from several international studies on RNMB. According to the available literature, this article is one of the few analyzing the patient’s demographic data, comorbidities, and the current clinical practice of intraoperative management of the neuromuscular blocking agents in a single predictive model for RNMB.

### Postoperative residual neuromuscular blockade

We showed that the incidence of RNMB in our study was 28.3%, i.e. approximately 1 out of every 3 patients under balanced general anaesthesia presented this complication. This figure still appears to be high today; however, it did not differ from the data from the most recent studies, where the incidence ranges from 14 to 32%^18–22^.

According to our results, RNMB was significantly decreased when intraoperative monitoring was performed and when rocuronium was reverted with sugammadex.

In cases where intraoperative NMM was used, the expected probability of RNMB decreased by a little over 25%. On the other hand, this probability decreased by 17% when neuromuscular management was done with rocuronium and sugammadex. However, more importantly, when we performed both techniques in the same intervention, the probability decreased by more than 30%.

Nevertheless, the incidence of RNMB in the group with sugammadex was 5.3% since it could probably be explained by clinical error. The dosing of sugammadex should be based on actual body weight^23–25^. For moderate NMB, defined as 1 to 2 twitches, the dose is 2 mg/kg, but for deep NMB, defined as a post-tetanic count of 1 to 2, it is up to 4 mg/kg, and if no monitoring is performed, the degree of NMB cannot be known. Many specialists routinely use 200 mcg of sugammadex^12^, which probably often leads to overdosing, but also to underdosing, particularly in patients weighing more than 100 kg^25^. The same occurs during emergency surgery. The dose of rocuronium is usually doubled, i.e. 1.2 mg/kg, when a rapid sequence intubation is used. Especially in these cases, as always, it is critical to use NMM and, if necessary, to use the correct dose of sugammadex per the patient’s real weight and degree of the blockade^24–27^.

### Postoperative pulmonary complication and intraoperative neuromuscular monitoring

Concerning the percentage of intraoperative NMM at our center, we found that in 30.3% of the surgical procedures, quantitatively monitoring of the NMB was used as routine clinical practice; this figure is similar to those of other studies and centers^7,12,13^. As stated by Naguib et al., the percentage of anaesthesiologists who rely solely on clinical signs for extubation remains very high^28^. According to our results, this lack of intraoperative NMM increased in both early and late POPC. However, this statement is only valid for desaturation and atelectasis since, in our sample, we have not been able to demonstrate that intraoperative NMM decreased the incidence of postoperative pneumonia and obstruction. The incidence for early POPC was 15.27%, and for late POPC was 8.12%, similar to those reported by Kheterpal et al. or Ledowsky et al.^3,29,30^.

### Postoperative pulmonary complication and pharmacological reversal

After analyzing the NMM, we should see what happened with the neuromuscular reversal and its influence on POPC. The controversy^31,32^ lies with some studies, such as Grosse-Sundrup et al.^2^, POPULAR^14^, or Li et al.^33^, which have reported that reversal, with one reversal agent and another, was not able to decrease these postoperative complications. Moreover, they questioned the utility of quantitative monitoring “the use of reversal agents or neuromuscular monitoring could not decrease this risk.”^14^.

We found that the use of rocuronium of sugammadex was associated with a lower risk of suffering early POPC in the PACU, which can be seen in Tables 2 and 3. In addition, when rocuronium and sugammadex were used instead of cisatracurium and neostigmine, the incidence of desaturation decreased by approximately 12% and, in the case of upper airway obstruction, by up to 2%.

Regarding the late POPC and pharmacological reversal, we also showed that the combination of rocuronium with sugammadex reduced them by up to 7%. In fact, the use of cisatracurium with neostigmine did not appear to decrease but subtly increase the incidence of these complications, which paradoxically aligned with the conclusions of recent studies^2,22,34–36^.

### Limitations

One of our limitations was that detection of late respiratory complications, both pneumonia and atelectasis, was based on clinical and laboratory criteria, and it may underestimate the complication rate. In addition, we reviewed the patient's clinical history without performing systematic X-ray in all cases since the patients can develop well-tolerated clinical postoperative atelectasis, implying unnecessary radiological exposure to all study patients. As described by Chen et al.^37^, a more effective and improved method for future research would be systematic examination with pulmonary ultrasound. It currently provides similar results to chest CT and chest X-ray for evaluating pneumonia and atelectasis^37,38^.

Moreover, our results were based on clinical management under real-life conditions. We have not analyzed other factors, such as mechanical ventilation parameters, recruitment maneuvers, opioid doses, fluid therapy, and others that are known to increase these complications and probably need to be assessed in subsequent studies^39,40^.

## Conclusion

Thus, based on the results of our study, it may be concluded that intraoperative NMM was one of the factors that most effectively reduced the risk of all these postoperative complications. Furthermore, the use of rocuronium with a pharmacological reversal with sugammadex was associated with a lower risk of RNMB and postoperative desaturation in the PACU and atelectasis during hospitalization.

## Data Availability

The datasets used and analyzed during the current study are available from the corresponding author on reasonable request.

## Abbreviations

RNMB: Residual neuromuscular blockade
POPC: Postoperative pulmonary complications
NMM: Neuromuscular monitoring
NMB: Neuromuscular blockade
PACU: Post-anaesthesia care unit
TOF: Train of four

## References

[1] Blobner M, Frick CG, Stauble RB (2015). Neuromuscular blockade improves surgical conditions (NISCO). Surg. Endosc..

[2] Grosse-Sundrup M, Henneman JP, Sandberg WS (2012). Intermediate acting non-depolarizing neuromuscular blocking agents and risk of postoperative respiratory complications: Prospective propensity score matched cohort study. BMJ.

[3] Kheterpal S, Vaughn MT, Dubovoy TZ (2020). Sugammadex versus neostigmine for reversal of neuromuscular blockade and postoperative pulmonary complications (STRONGER): A multi-center matched cohort analysis. Anesthesiology.

[4] Kim NY, Koh JC, Lee KY (2019). Influence of reversal of neuromuscular blockade with sugammadex or neostigmine on postoperative quality of recovery following a single bolus dose of rocuronium: A prospective, randomized, double-blinded, controlled study. J. Clin. Anesth..

[5] Togioka BM, Yanez D, Aziz MF, Higgins JR, Tekkali P, Treggiari MM (2020). Randomised controlled trial of sugammadex or neostigmine for reversal of neuromuscular block on the incidence of pulmonary complications in older adults undergoing prolonged surgery. Br. J. Anaesth..

[6] Bulka CM, Terekhov MA, Martin BJ, Dmochowski RR, Hayes RM, Ehrenfeld JM (2016). Nondepolarizing neuromuscular blocking agents, reversal, and risk of postoperative pneumonia. Anesthesiology.

[7] Martinez-Ubieto J, Ortega-Lucea S, Pascual-Bellosta A (2016). Prospective study of residual neuromuscular block and postoperative respiratory complications in patients reversed with neostigmine versus sugammadex. Minerva Anestesiol..

[8] Naguib M, Brull SJ, Kopman AF (2018). Consensus statement on perioperative use of neuromuscular monitoring. Anesth. Analg..

[9] Plaud B, Baillard C, Bourgain JL (2020). Guidelines on muscle relaxants and reversal in anaesthesia. Anaesth. Crit. Care Pain Med..

[10] Checketts MR, Alladi R, Ferguson K (2016). Recommendations for standards of monitoring during anaesthesia and recovery 2015: Association of Anaesthetists of Great Britain and Ireland. Anaesthesia.

[11] Stoelting RK (2016). Monitoring of neuromuscular blockade: What would you expect if you were the patient?. APSF Newslett..

[12] Hyman EC, Brull SJ (2017). Clarification: Current status of neuromuscular reversal and monitoring, challenges and opportunities. Anesthesiology.

[13] Todd MM, Hindman BJ, King BJ (2014). The implementation of quantitative electromyographic neuromuscular monitoring in an academic anesthesia department. Anesth. Analg..

[14] Kirmeier E, Eriksson LI, Lewald H (2019). Post-anaesthesia pulmonary complications after use of muscle relaxants (POPULAR): A multicentre, prospective observational study. Lancet Respir. Med..

[15] Mazo V, Sabaté S, Canet J (2014). Prospective external validation of a predictive score for postoperative pulmonary complications. Anesthesiology.

[16] Canet J, Hardman J, Sabaté S (2011). PERISCOPE study: Predicting post-operative pulmonary complications in Europe. Eur. J. Anaesthesiol..

[17] Kotake Y, Ochiai R, Suzuki T (2013). Reversal with sugammadex in the absence of monitoring did not preclude residual neuromuscular block. Anesthesia Analg..

[18] Cammu G (2020). Residual neuromuscular blockade and postoperative pulmonary complications: What does the recent evidence demonstrate?. Curr. Anesthesiol. Rep..

[19] Hristovska AM, Duch P, Allingstrup M, Afshari A (2018). The comparative efficacy and safety of sugammadex and neostigmine in reversing neuromuscular blockade in adults. A cochrane systematic review with meta-analysis and trial sequential analysis. Anaesthesia.

[20] Errando CL, Garutti I, Mazzinari G, Díaz-Cambronero Ó, Bebawy JF (2016). Residual neuromuscular blockade in the postanesthesia care unit: Observational cross-sectional study of a multicenter cohort. Minerva Anestesiol..

[21] Brueckmann B, Sasaki N, Grobara P (2015). Effects of sugammadex on incidence of postoperative residual neuromuscular blockade: A randomized, controlled study. Br. J. Anaesth..

[22] Fuchs-Buder T, Nemes R, Schmartz D (2016). Residual neuromuscular blockade: Management and impact on postoperative pulmonary outcome. Curr. Opin. Anaesthesiol..

[23] Merck & Co. Bridion^®^ (sugammadex) injection, for intravenous use: US prescribing information (2015) https://www.accessdata.fda.gov/drugsatfda_docs/label/2015/022225lbl.pdf (Accessed 14 Apr 2022).

[24] European Medicines Agency. Bridion^®^ (sugammadex) 100 mg/ml solution for injection: EU summary of product characteristics (2015) https://www.ema.europa.eu/en/medicines/human/EPAR/bridion (Accessed 14 Apr 2022).

[25] De Boer HD, Carlos RV, Brull SJ (2018). Is lower-dose sugammadex a cost-saving strategy for reversal of deep neuromuscular block? Facts and fiction. BMC Anesthesiol..

[26] Keating GM (2016). Sugammadex: A review of neuromuscular blockade reversal. Drugs.

[27] Alday E, Munoz M, Planas A (2019). Effects of neuromuscular block reversal with sugammadex versus neostigmine on postoperative respiratory outcomes after major abdominal surgery: A randomized-controlled trial. Canad. J. Anesth..

[28] Naguib M, Brull SJ, Johnson KB (2017). Conceptual and technical insights into the basis of neuromuscular monitoring. Anaesthesia.

[29] Ledowsky T, Hillyard S, O’Dea B, Archer R (2013). Introduction of sugammadex as reversal agent: Impact on the incidence of residual neuromuscular blockade and postoperative patient outcome. Indian J. Anaesth..

[30] Ledowski T, Szabó-Maák Z, Loh PS (2021). Reversal of residual neuromuscular block with neostigmine or sugammadex and postoperative pulmonary complications: A prospective, randomised, double-blind trial in high-risk older patients. Br. J. Anaesth..

[31] Unterbuchner C (2018). Neuromuscular block and blocking agents in 2018. Turk. J. Anaesthesiol. Reanim..

[32] Nemes R, Fülesdi B, Pongrácz A (2017). Impact of reversal strategies on the incidence of postoperative residual paralysis after rocuronium relaxation without neuromuscular monitoring: A partially randomised placebo controlled trial. Eur. J. Anaesthesiol..

[33] Li G, Freundlich RE, Gupta RK (2021). Postoperative pulmonary complications’ association with sugammadex versus neostigmine: A retrospective registry analysis. Anesthesiology.

[34] McLean DJ, Diaz-Gil D, Farhan HN, Ladha KS, Kurth T, Eikermann M (2015). Dose-dependent association between intermediate-acting neuromuscular-blocking agents and postoperative respiratory complications. Anesthesiology.

[35] Sasaki N, Meyer MJ, Malviya SA, Stanislaus AB, MacDonald T, Doran ME (2014). Effects of neostigmine reversal of nondepolarizing neuromuscular blocking agents on postoperative respiratory outcomes: A prospective study. Anesthesiology.

[36] Abad-Gurumeta A, Ripollés-Melchor J, Casans-Francés R (2015). A systematic review of sugammadex vs neostigmine for reversal of neuromuscular blockade. Anaesthesia.

[37] Chen Y, Zhang YG, Yi J (2022). Evaluation of postoperative residual curarisation after administration of neostigmine or sugammadex by diaphragmatic ultrasonography: A randomized double-blind controlled trial. Zhongguo Yi Xue Ke Xue Yuan Xue Bao.

[38] De la Quintana-Gordon FB, Nacarino-Alcorta B, Fajardo-Pérez M (2015). Basic lung ultrasound. Part 2. Parenchymal diseases. Rev. Esp. Anestesiol. Reanim..

[39] Colomina MJ, Ripollés-Melchor J, Guilabert P (2021). Observational study on fluid therapy management in surgical adult patients. BMC Anesthesiol..

[40] Ferrando C, Soro M, Unzueta C (2018). Individualised perioperative open-lung approach versus standard protective ventilation in abdominal surgery (iPROVE): A randomised controlled trial. Lancet Respir. Med..

